# A Dual-Cycle Isothermal Amplification Method for microRNA Detection: Combination of a Duplex-Specific Nuclease Enzyme-Driven DNA Walker with Improved Catalytic Hairpin Assembly

**DOI:** 10.3390/ijms26020689

**Published:** 2025-01-15

**Authors:** Yu Han, Shuang Han, Ting Ren, Liu Han, Xiangyu Ma, Lijing Huang, Xin Sun

**Affiliations:** School of Pharmaceutical Sciences, Jilin Medical University, Jilin 132013, China

**Keywords:** duplex-specific nucleases, microRNAs, catalytic hairpin assembly, DNA walker

## Abstract

The association between microRNAs and various diseases, especially cancer, has been established in recent years, indicating that miRNAs can potentially serve as biomarkers for these diseases. Determining miRNA concentrations in biological samples is crucial for disease diagnosis. Nevertheless, the stem-loop reverse transcription quantitative PCR method, the gold standard for detecting miRNA, has great challenges in terms of high costs and enzyme limitations when applied to clinical biological samples. In this study, an isothermal signal amplification method based on a duplex-specific nuclease (DSN) enzyme-driven DNA walker and an improved catalytic hairpin assembly (CHA) was designed for miRNA detection. First, biotin–triethylene glycol-modified trigger-releasable DNA probes were conjugated to the streptavidin-coated magnetic beads for recognizing the target miRNA. The DSN enzyme specifically hydrolyzes DNA strands when the DNA probe hybridizes with the targeted miRNA. This recycling process converts the input miRNA into short trigger fragments (catalysts). Finally, three hairpins of improved CHA are driven by this catalyst, resulting in the three-armed CHA products and a fluorescence signal as the output. This dual-cycle biosensor shows a good linear relationship in the detection of miR-21 and miR-141 over the final concentration range of 250 fM to 50 nM, presenting an excellent limit of detection (2.95 amol). This system was used to detect miR-21 and miR-141 in MCF-7 and 22RV1 cells, as well as in 1% human serum. This system can be used to evaluate the expression levels of miRNAs in different biological matrices for the clinical diagnosis and prognosis of different cancers.

## 1. Introduction

MicroRNAs (miRNAs) are a category of biomarkers implicated in a wide range of disorders, particularly cancer [1,2]. For instance, there is a correlation between the occurrence of cervical and ovarian malignancies and the abnormal expression of miR-21 [3]. Further, in patients diagnosed with gastric carcinoma, serum miR-let-7a levels were downregulated [4]. In contrast, the serums of individuals with papillary thyroid carcinoma present increased miR-146b-5p levels and decreased miR-199b-5p levels [5,6]. Moreover, miRNAs exist not only in tissues but also in biological fluids [2]. The aforementioned studies have demonstrated the critical role of miRNAs in cancer diagnosis and treatment. However, the determination of miRNA in various biological samples is hindered by several factors, mostly because of their unique features [7]. Currently, stem-loop reverse transcription quantitative PCR is considered the gold standard for assessing miRNA expression levels [8]. Although this method has been widely used in analytical chemistry and life sciences, it typically involves a two-step process that can lead to operational challenges, high polymerase expenses, and long analysis time [9]. Therefore, a novel strategy with a high amplification efficiency, low background signal, and excellent specificity is urgently required.

It is known that nucleic acids (DNA and RNA) are a class of biomacromolecules that carry genetic information and play a significant role in biological growth and regular operations. Owing to the rapid developments in life sciences and analytical chemistry in recent years, the powerful potential of analytical methods in nucleic acid detection, both in vitro and in vivo, has been recognized [10]. Further, as an isothermal amplification method for nucleic acids, nucleic acid sequence-based amplification has attracted considerable interest. In particular, enzyme-free nucleic acid circuits overcome the shortcomings of traditional miRNA detection methods. For example, processes such as catalytic hairpin assembly (CHA) and hybridization chain reaction (HCR) are driven by toehold-mediated strand displacement and branch migration [11,12]. Moreover, their reaction conditions are mild, and they could be easily combined with other amplification technologies. DNA walkers, usually based on DNAzymes or CHA on the surface of magnetic microparticles, have been proposed and applied in miRNA detection [13,14,15]. Nevertheless, the prevailing methods for DNA walking machines face ongoing difficulties owing to their inadequate analytical sensitivity, necessitating the use of complicated signal amplification approaches [16]. Thus, to enhance the sensitivity of detection, the traditional DNA walking mechanism facilitated by the self-assembly of DNA was changed to a DNA walker driven by the duplex-specific nuclease (DSN) enzyme. DSN enzyme-assisted signal amplification (DSNSA) approaches are receiving increasing attention for miRNA and protein detection [17,18,19,20,21]. This method utilizes a class of DNases derived from the Kamchatka crab, which specifically hydrolyze double-stranded DNA (dsDNA) or DNA in DNA/RNA hybrids while maintaining the single strand intact [22]. Therefore, only a signal DNA sequence needs to be designed to recognize the target miRNA, which decreases detection costs and the risk of nonspecific hydrolysis. A signal output is often used in high-performance liquid chromatography (HPLC), coupled mass spectrometry (MS), and electrochemical methods based on DSNSA strategies [23,24]. However, these methods have intrinsic limitations, such as being time-consuming (>12 h) and requiring specialized equipment. The CHA is a straightforward procedure with excellent specificity. However, CHA suffers in terms of its reaction efficiency and background leakage. Recently, Kim et al. attempted to reduce the signal leakage problem of CHA and established an improved CHA driven by a 30 bp DNA sequence [11].

Considering these findings, we devised a novel strategy that utilizes a DSN enzyme-driven DNA walker and an improved CHA approach, termed as a dual-cycle isothermal amplification method. A single-stranded biotin–triethylene glycol (TEG)-modified catalyst-released DNA probe was designed to recognize the target miRNA. This probe was conjugated to the surface of streptavidin-coated magnetic beads (SA-MBs). As the DSN enzyme hydrolyzes the DNA, the target miRNA is released, and it binds to a new DNA probe on the bead. This mechanism is similar to that of a DNA walker, in which the released miRNA maintains the specificity of the DSN enzyme and accumulates the trigger fragments (catalyst). Subsequently, this catalyst recognizes the toeholds of hairpin 1 (HP1) and alters the secondary conformation of DNA, producing double-stranded DNA products. HP2 and HP3 are also opened by this product to form a three-armed CHA product (3-CHA). Finally, the catalyst is released, and it continually recognizes the toeholds of HP1. This process enables the swift and accurate analysis of miRNAs, leading to exceptional sensitivity and specificity. Moreover, it effectively mitigates the effects of biological matrices, thus offering a robust detection methodology for the biochemical investigation of miRNAs.

## 2. Results

### 2.1. Design Principle of the Combination of a DSN-Driven DNA Walker with Improved CHA for miRNA Detection

The miRNA detection method developed in this study involves two major isothermal signal amplification methods: DSN-mediated isothermal signal amplification (DSNSA) and CHA. In simple terms, the initial miRNA input is amplified by the short-chain DNA fragments formed in the first reaction cycle. Then, the DNA fragment triggers the CHA reaction in the second cycle, resulting in fluorescence resonance energy transfer and a corresponding fluorescence signal that is used to quantify the target miRNA. Our group previously published a study on the detection of miR-146b utilizing a combination of DSNSA and CHA reactions [5]. Although this approach has excellent specificity, its detection capability is limited to the picomolar (pM) levels. The reasons for this limitation are the poor efficiency of DSNSA and, more significantly, the substantial background leakage of the CHA reaction. Therefore, to enhance the detection potential of this method, we immobilized the DNA probe on SA-MBs and activated the DSN enzyme to hydrolyze the DNA probe along the bead surface, resulting in the production of short-chain DNA fragments. The improved CHA has a high signal-to-noise ratio, which is driven by the short-chain DNA fragments.

**Scheme 1:**
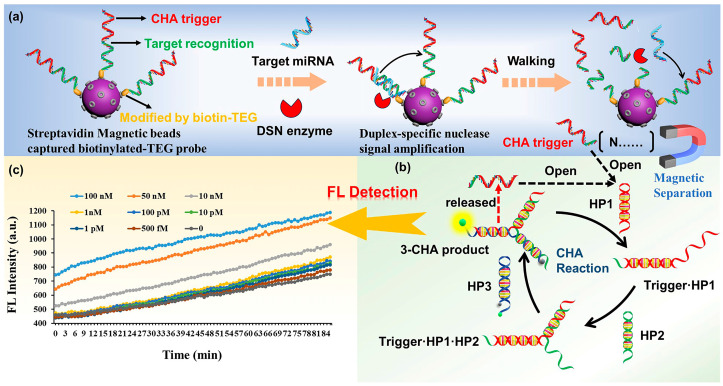
Schematic of the combination of a duplex-specific nuclease (DSN)-driven DNA walker with improved catalytic hairpin assembly (CHA). (**a**) A DSN-driven DNA walker was designed to accumulate the trigger fragments; (**b**) the improved CHA was triggered, and fluorescence resonance energy transfer was utilized for signal detection; (**c**) the change of the fluorescence signal occurred within 85 min.

The principle of this reaction is illustrated in Scheme 1. The biotinylated TEG oligonucleotide sequence was designed in two parts. One part (green in Scheme 1) was designed to recognize the target miRNA at the 3′-end. The 3′-end of the DNA probe was modified with biotin–TEG, which was used to load the probe on the surface of SA-MBs. When the DSN enzyme was added to the reaction solution, the sequence shown in red in Scheme 1 was hydrolyzed and the target miRNA was simultaneously released. The other part of the nucleotide sequence comprised the catalyst sequence that triggers the CHA reaction, as shown in red at the 5′-end. When the solution contained only the catalyst, it successively recognized the toehold of the hairpin primers and formed an assembly complex of double strands (3-CHA). The catalyst was then released back into the solution, and it continually self-assembled with hairpin primers to form 3-CHA. One of the hairpin primers was modified with FAM and BQH1. When the 3-CHA product was generated, the conformation of the original primer was altered, leading to the separation of the fluorescent and the quenching groups. This generated a strong fluorescent signal from the 3-CHA product. In this study, a normalization method was used to determine the optimal conditions and calculated as follows: ΔF = (F_Sample_ − F_Blank_)/F_Blank_ × 100%, where F_Blank_ refers to the background signal in the absence of target miRNA, and F_Sample_ refers to the recorded signal following the addition of miRNA to the mixture. When quantifying miRNA using this approach, we measure the difference in fluorescence intensity, which is calculated by subtracting the fluorescence intensity of the blank from the fluorescence intensity of the sample.

### 2.2. Verification of the Feasibility of the Detection System

**Figure 1:**
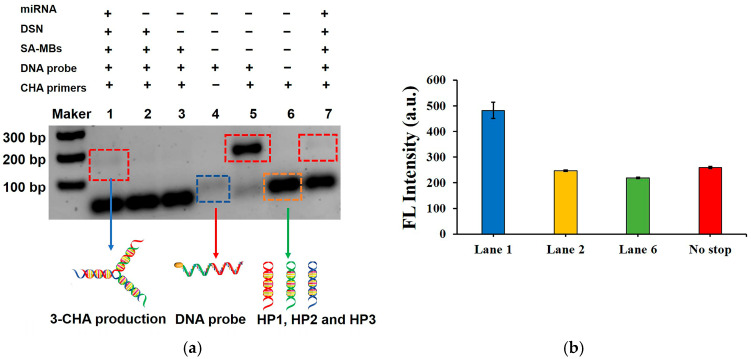
(**a**) Agarose gel electrophoresis of the newly developed miRNA detection method. Lane 1: miRNA + DSN + SA-MBs + DNA probe + CHA primers+ 2× DSN stop solution; lane 2: DSN + SA-MBs + DNA probe + CHA primers; lane 3: SA-MBs + DNA probe + CHA primers; lane 4: DNA probe; lane 5: DNA probe + CHA primers; lane 6: CHA primers; lane 7: miRNA + DSN + SA-MBs + DNA probe + CHA primers; (**b**) fluorescence signals of the corresponding solutions under the same conditions (error bars: SD; *n* = 3).

To verify the feasibility of the newly developed miRNA detection approach, the reaction solution was subjected to agarose gel electrophoresis. As shown in Figure 1a, when the target miRNA, DSN enzyme, DNA probe, SA-MBs, and the three HPs of CHA were present in lane 1, an extra band (marked with a red frame) with a molecular weight of approximately 200 bp, corresponding to 3-CHA products, was observed. A bright band of 3-CHA products was also observed when the DNA probe and three HPs of CHA were present simultaneously (lane 5). Hence, the additional band observed in lane 1 can be attributed to the activity of the DSN enzyme, which hydrolyzed the DNA probe attached to the magnetic beads, producing shorter DNA fragments. After magnetic separation, the DNA fragments drive the CHA reaction to generate 3-CHA products. The 3-CHA products were not observed in lane 2 without the miRNA and in lanes 3 and 6 without the miRNA and DSN, because the absence of the DSN enzyme and miRNA prevented the activation of the CHA reaction involving the three primers. Typically, when working with DSN enzymes, an inactivation process is performed following the enzymatic reaction. However, it is important to note that this method always prolongs the detection time and requires extra processing steps. When the DSN enzyme reaction solution that was not inactivated was mixed with the CHA primer, the 3-CHA products appeared on the agarose gel, indicating that the activity of the DSN enzyme was not affected in this detection system. The feasibility of the reaction was also confirmed through fluorescence-based detection. As shown in Figure 1b, the fluorescence intensity of the solution containing only the three HPs of CHA was poor (green bar). In the absence of the target miRNA (yellow bar), the fluorescence intensity is equivalent to that observed in lane 6. The fluorescence intensity of 3-CHA was significantly enhanced only when the target miRNA, DSN enzyme, DNA probe, SA-MBs, and three HPs of CHA were present simultaneously (shown as the blue bar). In the absence of DSN enzyme stop solution (red bar), the fluorescence intensity was slightly higher than those of the reactions corresponding to lanes 2 and 6 (yellow and green bars). The observation of nonspecific fluorescence signals may be attributed to the hydrolysis of the double-labeled HP molecules by the DSN enzyme. Hence, to improve the sensitivity and specificity of miRNA detection, a stop solution should be added at the end of the DSN-mediated reaction. Thus, the results of agarose gel tests and fluorescence detection suggest that a sensitive analysis of the target miRNA may be achieved using the DSN-driven DNA walker and improved CHA-mediated isothermal signal amplification.

### 2.3. Optimization of the Reaction Conditions for miRNA Determination

**Figure 2:**
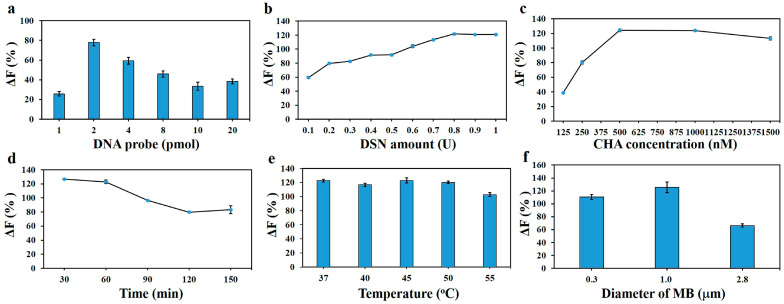
Optimization of experimental conditions: (**a**) amount of the DNA probe; (**b**) DSN dose; (**c**) CHA concentration; (**d**) reaction time and (**e**) temperature of the DSN enzyme-driven DNA walker; (**f**) diameters of magnetic beads (error bars: SD; *n* = 3).

To obtain the best sensitivity in the detection of target miRNAs, we conducted optimization experiments, in which the amount of the DNA probe, DSN dose, the amounts of the three HPs of CHA, the DSN reaction time and temperature, and the diameter of SA-MBs were varied. ΔF%, which represents the ratio of fluorescence intensity of the reaction solution with and without the target miRNA, was used for optimization. As shown in Figure 2a, the ΔF% reached the maximum when 2 pmol of the DNA probe was used. The ΔF% value gradually decreased as the amount of the DNA probe exceeded 4 pmol; therefore, 2 pmol was selected as the optimal amount of the DNA probe. As shown in Figure 2b, the ΔF% value increased with increasing DSN dose and plateaued as the DSN dose reached 0.8 U. This result indicates that the reaction efficiency of the 0.8 U DSN on the surface of the bead was the highest. Further, as shown in Figure 2c, the ΔF% plateaued when the amount of the three HPs of CHA was increased to 500 nM, indicating that the background signal leakage at this HP concentration was the least. Then, the effects of the reaction time and temperature of the DSN reaction on the ΔF% value were studied. As shown in Figure 2d,e, the best experimental conditions were 30 min and 37 °C. Next, the reaction efficiency of the DSN on the surface of magnetic beads with varying diameters was investigated. As shown in Figure 2f, the ΔF% reached the maximum when 1.0 μm diameter SA-MBs were used. Finally, the influence of incorporating a neutral DNA spacer on the detection capability of this strategy was evaluated. As shown in Figure S1, the results of using a neutral spacer are consistent with those of previous studies, indicating that the addition of a neutral DNA spacer enhances both the specificity and stability of hybridization [25,26].

### 2.4. Performance and Selectivity of the Detection Strategy

**Figure 3:**
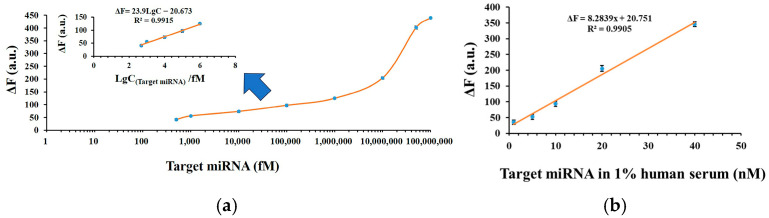
Calibration curve of the novelty developed system for miR-141 detection. (**a**) Linear correlation between ΔF and the logarithm of miR-141 concentration (500 fM to 1 nM); (**b**) calibration curve for miRNA detection in 1% human serum. ΔF represents the fluorescence intensity of the sample subtracted from that of the blank (error bars: SD; *n* = 3).

Under optimal reaction conditions, a linear regression curve was obtained using standard solutions with different concentrations of miR-21 and miR-141. For this, ΔF, which represents the difference in the fluorescence intensity of the sample and blank groups, was used (the reaction solution without the addition of target miRNA as the blank group). As shown in Figure 3a, a strong linear relationship between the fluorescence intensity and the logarithm of miR-141 concentration was observed in the range of 500 fM to 1 nM. The limit of detection (LOD) was calculated to be 118 fM (2.95 amol) by the standard 3σ. Further, this approach provided a similar detection capability in the detection of miR-21 (see Figure S2). In addition, we used only CHA in our approach to trigger chain detection, and the results are displayed in Figure S3. When only CHA was utilized, the detection limit for the target analyte was 500 pM (12.5 fmol). However, when the DSN-driven DNA walker and CHA were combined, the target analyte could be detected over a wider range of concentrations. This result indicates that this technique successfully combines the two isothermal signal amplification methods. Table S1 summarizes some results published in recent years on the detection of nucleic acids (RNA or DNA) utilizing DNA walking machines [15,27,28,29,30,31,32,33,34,35]. As shown in Table S1, the LOD of the previous studies was poorer than that achieved in this study. Thus, the designed dual-cycle isothermal amplification system is a powerful tool for miRNA detection.

As shown in Figure S4, the specificity of the developed novelty system was investigated for off-target miRNAs (miR-375, miR-221, and miR-210). The fluorescence intensity of these is no more than 8% of that of miR-21. To evaluate the applicability of this system for clinical disease diagnosis, human serum was extracted without using a reagent kit and diluted to 1% with DSN master buffer. Standard solutions with different miR-141 concentrations in the 1 nM to 40 nM range were added to the diluted serum samples. Figure 3b plots the ΔF value as a function of the concentration of the miR-141 standard solution in a 1% serum matrix with different substance masses. In this case, ΔF represents the difference between the fluorescence intensities of the sample and blank groups, and a serum solution without target miRNA was used as the blank group. Thus, the strategy developed in this study could directly detect miR-141 in serum and overcome the effects of the serum matrix, thus eliminating the need to extract total RNA using a reagent kit, which saves cost and time, and laying the foundation for the accurate analysis of the miR-141 concentration in human serum samples.

### 2.5. Analysis of miRNAs in Real Samples

**Figure 4:**
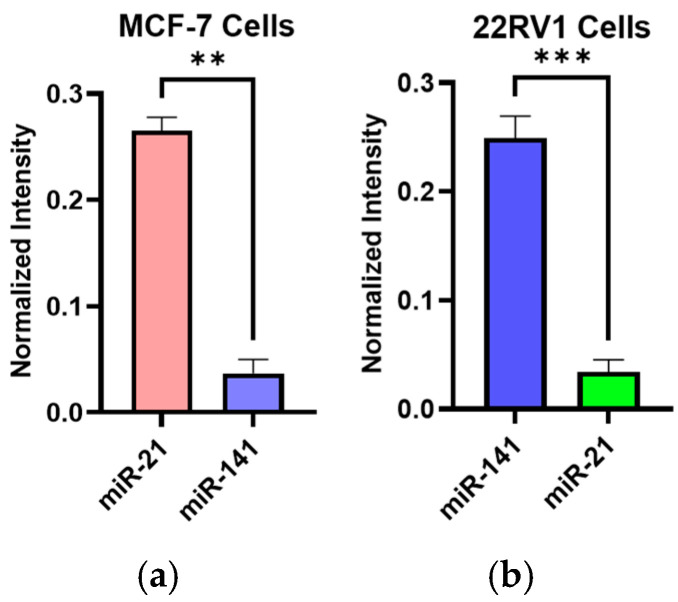
Application of this developed method to detect miR-141 in the clinical samples. (**a**) Detection of total RNA in MCF-7 cells (** *p* < 0.01); (**b**) detection of total RNA in 22RV1 cells (*** *p* < 0.001) (error bars: SD; *n* = 3).

Following the extraction of total RNA from cells using a kit, the quantity of RNA was measured using Nanodrop One. As shown in Figure S5a,b, the A260/A280 ratio of the extracted total RNA passed the standards and was suitable for quantitative analysis. Figure 4a,b present the total RNAs in MCF-7 and 22RV1 cells. The results indicate that the level of miR-21 expression in MCF-7 cells was significantly higher than that of miR-141 (*p* < 0.01). On the other hand, the level of miR-141 expression in 22RV1 cells was significantly higher than that of miR-21 (*p* < 0.001). This result is consistent with the conclusions of previously published studies [36,37]. Thus, our system can potentially be used in practical clinical malignant tumor differentiation and biological research.

## 3. Discussion

In this study, a detection method for miRNA was developed by combining the DSN enzyme-assisted signal amplification mediated by DNA walking with the catalytic hairpin assembly (CHA). The results demonstrated that this method could resist the serum matrix, thus having a promising application prospect in the subsequent miRNA quantification of human serum from prostate cancer patients.

As shown in Table S1, some methods related to DNA walking, DSN enzyme, and CHA for detecting miRNA were summarized. After optimization, the reaction time of the method developed in this study was significantly shorter than that of other methods. Especially for the use of electrochemical methods, it usually takes a long time to prepare electrodes or gold nanoparticles [28]. Although electrochemical methods have demonstrated excellent detection capabilities, they are not suitable for the analysis of many clinical samples. In addition, we found that the detection sensitivity was more excellent when the DSN enzyme was used in combination with other biological enzymes. However, it also increased the detection cost and the complexity of the operation at the same time.

Finally, in this study, we used prostate cancer cell lines to verify the feasibility of the new method in miR-21 and miR-141 detection. Moreover, through reading the literature [38,39,40], it was found that there might be simultaneous dysregulation of the expression of miR-21 and miR-141 in the serum of prostate cancer patients. Hence, it is more meaningful to develop detection methods for simultaneously detecting multiple miRNAs in the follow-up work.

## 4. Materials and Methods

### 4.1. Reagents and Apparatus

DSN enzyme, the master buffer of DSN enzyme, and DSN enzyme stop solution were, respectively, obtained from Genstone Biotech Co., Ltd. (Beijing, China) and Shenzhen Newborn Co., Ltd. (Shenzhen, China). The SA-MBs of 300 nm, 1.0 μm, and 2.8 μm diameters were bought from BioMag Scientific Inc. (Wuxi, China). A DNA ladder was bought from ServiceBio Technology Co., Ltd. (Wuhan, China). Precast agarose gel (2%), ultrapure water (DNase/RNase-free, sterile), diethylpyrocarbonate (DEPC)-treated water (DNase/RNase-free, sterile), Tween-20, 3 M KCl, 1 M Tris-HCl, and 5 M NaCl were, respectively, obtained from Beyotime Biotechnology Co., Ltd. (Shanghai, China). The above reagents and ultrapure water were used to prepare the binding solution and the washing buffer. They, respectively, consisted of 20 mM Tris-HCl, 300 mM NaCl, 5 mM KCl, pH 8.0, and 7.0 mM Tris-HCl, 130 mM NaCl, 0.05% Tween-20, pH 8.0. The buffer solution used for CHA consisted of 20 mM Tris-HCl, 300 mM NaCl, and 5 mM KCl. All of the DNA hairpins and probes were synthesized and purified by Sangon Biotech Co., Ltd. (Shanghai, China). All miRNAs used in this study were synthesized by Shanghai GenePharma Co., Ltd. (Shanghai, China) and diluted with DEPC-treated water. Their base sequences of the miRNAs are listed in Table S2.

Nanodrop one (Thermo Fisher Scientific Inc., Waltham, MA, USA) was utilized to quantify all standard DNA oligonucleotides and miRNA solutions. The DSN-driven DNA walker and SA-MB-conjugated DNA probe were prepared using a constant temperature oscillator (DLAB Scientific Co., Ltd., Beijing, China). The fluorescence of the 3-CHA products was monitored using an Infinite E Plex multimode microplate reader (Tecan Trading AG, Männedorf, Switzerland).

### 4.2. Synthesis of SA-MBs Conjugated Biotin–TEG-Modified Trigger-Released DNA Probes

First, 2 μL of SA-MBs (10 μg) was washed three times with washing buffer. Then, 100 μL of the biotin–TEG-modified catalyst-released DNA probe (20 nM) and 10 μg of SA-MBs were mixed and incubated at 37 °C for 60 min. Finally, the supernatant was discarded after magnetic separation, and the SA-MB-conjugated DNA probe was washed again with washing buffer three times. This washing was performed to avoid excess DNA probe remaining in the tube. The supernatant was utilized to record the OD260 value used to calculate the binding rate.

### 4.3. Combining the DSN-Driven DNA Walker and Improved CHA to Detect the Targeted miRNA

The prepared SA-MB-conjugated DNA probe, 25 μL of different amounts of miRNA, and 0.016 U/μL of DSN were mixed in master buffer. The DSN-driven DNA walker reaction was performed on a constant-temperature oscillator, and the resulting sample was incubated for 30 min at 37 °C. Thereafter, the beads were magnetically separated, and the supernatant was added to 20 μL of the DSN enzyme stop solution (10 mM EDTA) at room temperature to inactivate the DSN enzyme. Finally, 12.5 μL of the supernatant was added to a buffered stop solution mixed with the three HPs of CHA (0.5 μM). The fluorescence signal of the resultant CHA product was monitored for 85 min at 25 °C in a microplate reader and recorded at 522 nm under 494 nm excitation. The normalized fluorescence intensity (*F*/*F*_0_ − 1) was used to optimize the reaction conditions and quantify the miRNA.

### 4.4. Agarose Gel Electrophoresis

A mixed solution consisting of 2.5 pmol of the target miRNA, 0.8 U of the DSN enzyme, 6.25 pmol of the three HPs of CHA, and 2.5 pmol of the DNA probe was prepared following the experimental procedure outlined in Section 4.3. Then, 10 μL of the sample solution (containing 9 μL of the reaction solution and 1 μL of 10× loading buffer) and the loading solution were added to the prepared 2% agarose gel. The power was turned on, and the voltage was adjusted to 150 V. When the blue band moved to 2/3 of the gel, electrophoresis was stopped, and the gel was analyzed using an Image Quant 800 imaging system (Cytiva, Marlborough, MA, USA).

### 4.5. Extraction of Total RNA from MCF-7 and 22RV1 Cells and Human Serum Collection

MCF-7 and 22RV1 cells were cultured in T25 flasks. Then, the total RNAs of these cells were extracted using the miRcute miRNA Isolation Kit, according to the manufacturer’s instructions. Serum from a healthy human volunteer (29 years, male) was diluted to 1% with 1× DSN buffer for subsequent use. All experiments were performed in accordance with the guidelines of the Declaration of Helsinki and were approved by the Ethics Committee (No.: 2024031505) of Jilin Medical College Affiliated Hospital.

### 4.6. Statistical Analysis

Statistical analyses were performed using the paired *t*-test. A *p*-value of <0.05 (0.001) was considered statistically significant.

## 5. Conclusions

In this study, we developed a novel dual-cycle signal amplification system by combining a DSN-driven enzyme walker and an improved CHA process for highly sensitive and specific miRNA detection in clinical biological samples. To evaluate the feasibility of this system, miR-141 and miR-21 were used as model analytes. The developed system relied on two signal amplification methods. On the one hand, a DNA probe was loaded on the surface of SA-MBs, and the DSN enzyme was added to hydrolyze the DNA probe and achieve the self-circulation of the target miRNA. The accumulated DNA fragments (catalysts) represent the amount of the target miRNA in the investigated system. On the other hand, the catalyst improved the CHA reaction and formed 3-CHA products with modified FAM and BQH1. The CHA process induced fluorescence resonance energy transfer, resulting in a fluorescence signal that was measured to quantify the target miRNA. This dual amplification system exhibits a good linear relationship in the range of 500 fM to 100 nM for the targeted miRNA and excellent selectivity against off-target miRNAs. This study proposes an efficacious miRNA detection approach for cancer prognostics and diagnostics, along with a novel strategy integrating DSN enzymes and enzyme-free amplification.

## Data Availability

Data are contained within the article and Supplementary Materials.

## Supplementary Materials

The following supporting information can be downloaded at https://www.mdpi.com/article/10.3390/ijms26020689/s1.
