# Supplementary material for: A Dual-Cycle Isothermal Amplification Method for microRNA Detection: Combination of a Duplex-Specific Nuclease Enzyme-Driven DNA Walker with Improved Catalytic Hairpin Assembly
Source: Int J Mol Sci. 2025 Jan 15;26(2):689. doi: 10.3390/ijms26020689 (PMC11766441; doi:10.3390/ijms26020689)
Supplement: Supplementary file 1 [file ijms-26-00689-s001.zip › ijms-3402922-supplementary.pdf]

Supplementary Materials

A Dual-Cycle Isothermal Amplification Method for microRNA Detection: Combination of a Duplex-Specific Nuclease Enzyme-Driven DNA Walker with Improved Catalytic Hairpin Assembly

Yu Han[1,*], Shuang Han[1], Ting Ren[1], Liu Han[1], Xiangyu Ma[1], Lijing Huang[1] and Xin Sun[1,*]

1 School of Pharmaceutical Sciences, Jilin Medical University, Jilin 132013, China

* Correspondence: hanyu.jlmu@vip.163.com (Y.H.); sunxinbh@126.com (X.S.)

Table of Content

Table and Figure 3

Table and Figure

Table S1. Comparison of the analytical performance of the novel developed method with DNA walking machine, DSN enzyme-assisted signal amplification, and catalytic hairpin assembly methods for miRNA detection.

Target analyte	Reaction time [a]	Enzyme	Combined with other methods [b]	Detection range	Limit of detection	Refs
miRNA-122	120 min				1 nM	15
miR-21	215 min	Duplex-specific nuclease and Bst DNA polymerase	Primer exchange reaction	100 fM-50 nM	67. 771 fM	27
miR-141	85 min	Duplex-specific nuclease	Toehold-mediated strand displacement reaction	0.001-100 nM	0.97 pM	28
miR-21	120 min	Duplex-specific nuclease	3D nanochannel sensor	1 fM-1 nM	1 fM	29
miRNA-122	240 min	Enzyme-free		0.156-7 nM	42.94 pM	30
miRNA-21	120 min	Enzyme-free		0.05-20 nM	41 pM	31
miR-let-7a	110 min	Duplex-specific nuclease and Cas12a	CRISPR-Cas12a	100 fM-500 nM	64.17 fM	32
miRNA-203a	120 min	Enzyme-free		0.1-150 nM	74 pM	33
miRNA-21	30 min	Enzyme-free	Hybridization chain reaction	0.025-1 nM	1.8 pM	34
miRNA-141	40 min	Enzyme-free	DNA-templated silver nanocluster	0-200 nM	297.1 pM	35
miRNA-141	35 min	Duplex-specific nuclease		500 fM-100 nM	118 fM	This method

a: It refers to the reaction time of the target miRNA with primers and enzymes.

b: Other methods are those except for DNA walking machine, DSN enzyme-assisted signal amplification and catalytic hairpin assembly.

Table S2. The sequences of DNA and RNA used in the study are as follows (5′-3′).

Name	Sequence (5' – 3')
Biotion-TEG DNA probe-21	CTACACCAATTCACCATCCTTAGCACTTCGTCAACATCAGTCTGAT AAGCTA-Biotion-TEG
Biotion-DNA probe-21	CTACACCAATTCACCATCCTTAGCACTTCGTCAACATCAGTCTGAT AAGCTA-Biotion
Biotion-TEG DNA probe-141	CTACACCAATTCACCATCCTTAGCACTTCGCCATCTTTACCAGACA GTGTTA-Biotion-TEG
HP1	CGAAGTGCTAAGGATGGTGAATTGGTGTAGACTTCGGCTACACTA CACCAATTCACCATCCTTAGC
HP2	GATGGTGAATTGGTGTAGTGTAGCCGAAGTACCATCCTTAGCACT TCGGCTACACTACACCAATTC
HP3	BQH1-GTGTAGTGTAGCCGAAGTGCTAAGGATGGTCTACACCAATTCACC ATCCTTAGCACTTCGGCTACA-FAM
mi-RNA-21	UAGCUUAUCAGACUGAUGUUGA
mi-RNA-375	UUUGUUCGUUCGGCUCGCGUGA
mi-RNA-221	AGCUACAUUGUCUGCUGGGUUUC
mi-RNA-210	CUGUGCGUGACAGCGGCUGA
mi-RNA-141	UAACACUGUCUGGUAAAGAUGG

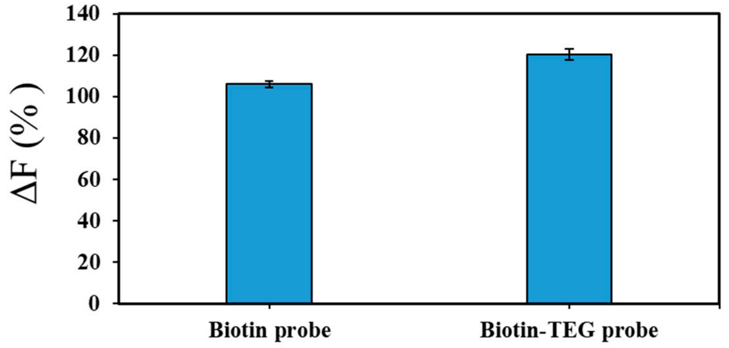

Figure S1. The different DNA probes influencing the newly developed method.

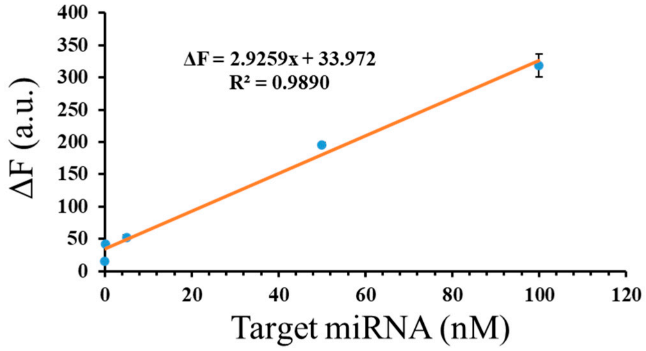

Figure S2. Calibration curve of the novelty developed system in the range from 500 fM to 100 nM for miR-21 detection.

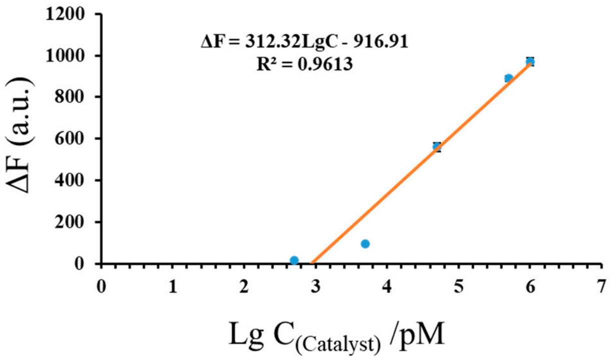

Figure S3. Calibration curve of the improved catalytic hairpin assembly in the range from 500 pM to 1 µM for catalyst detection.

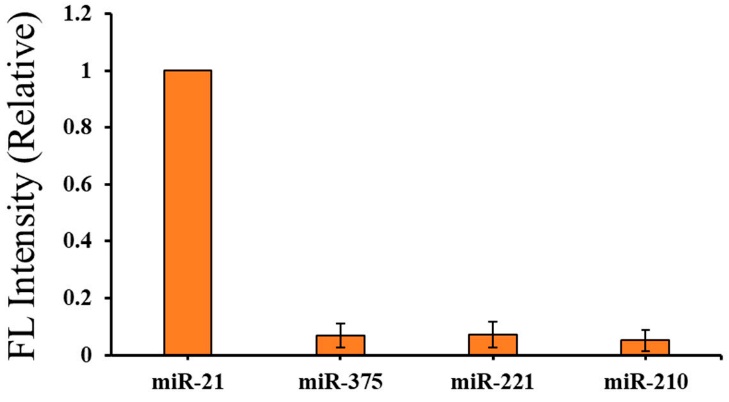

Figure S4. Specificity of the novelty developed strategy.

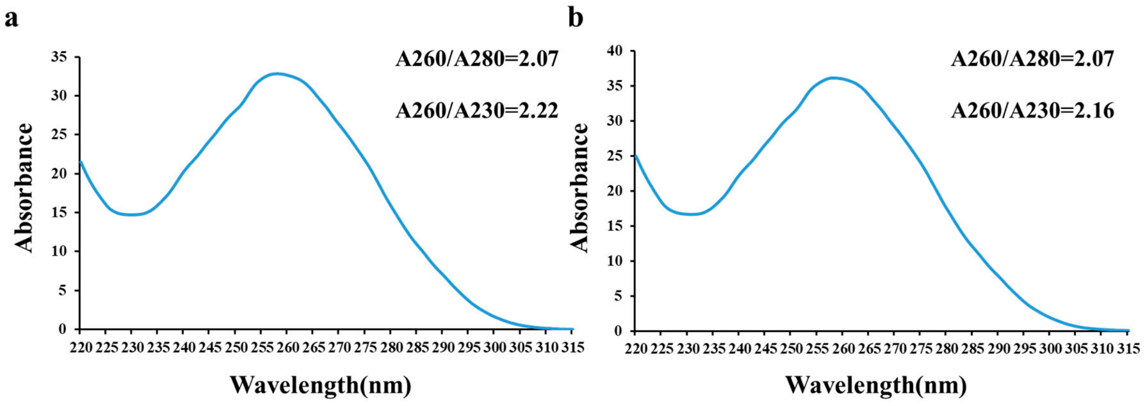

Figure S5. Absorbance spectra from total RNAs utilized the miRcute miRNA Isolation Kit. (a) MCF-7 cells. (b) 22RV1 cells.

Reference:

[15] C. Jung, P.B. Allen, A.D. Ellington, A stochastic DNA walker that traverses a microparticle surface, Nat. Nanotechnol. 11 (2016) 157-163.

[27] Bai, D.; Zhang, Y.Y.; Pu, J.; Zhang, L.; Yu, H.Y.; Han, X.L.; Lv, K.; Wang, L.; Li, J.J.; Tang, A.; et al. Sequence-Unconstrained DNA Computing: DSN cycling and PER circuitry for dynamic miRNAs analysis and multifunctional logic operations. *Chem. Eng. J.* **2024**, *499*, 156045.

[28] Yu, L.Y.; Peng, Y.; Sheng, M.T.; Wang, Q.; Jin, Z.Y.; Huang, J.S.; Yang, X.R. Electrochemical biosensing platform based on toehold-mediated strand displacement reaction and DSN enzyme-assisted amplification for two-target detection. *ACS Appl. Mater. Interfaces* **2024**, *16*, 45695-45703.

[29] Liao, T.B.; Luo, K.X.; Tu, J.Y.; Zhang, Y.L.; Zhang, G.J.; Sun, Z.Y. DSN signal amplification strategy based nanochannels biosensor for the detection of miRNAs. *Bioelectrochemistry* **2024**, *160*, 108771.

[30] Q. Li, X.H. Liang, X.M. Mu, L. Tan, J.N. Lu, K. Hu, S.L. Zhao, J.N. Tian, Ratiometric fluorescent 3D DNA walker and catalyzed hairpin assembly for determination of microRNA, Microchim. Acta 187 (2020) 365.

[31] T.Y. Yang, J. Fang, Y.C. Guo, S.C. Sheng, Q.L. Pu, L. Zhang, X.Y. Ou, L. Dai, G.M. Xie, Fluorometric determination of microRNA by using an entropy-driven three-dimensional DNA walking machine based on a catalytic hairpin assembly reaction on polystyrene microspheres, Microchim. Acta 186 (2019) 574.

[32] Qin, H.J.; Chen, Z.Y.; Zuo, F.J.; Cao, R.F.; Wang, F.Y.; Wu, H.P.; Wang, S.J.; Xie, Y.J.; Ding, S.J.; Min, X. "DSN-mismatched CRISPR" sensor for highly selective and sensitive detection of under-expressed miR-let-7a. *Anal. Chim. Acta* **2024**, *1295*, 342273.

[33] P. Yang, Q.J. Zhu, Z.P. Chen, Z.Z. Yang, R. Yuan, Y. Li, W.B. Liang, A target-initiated autocatalytic 3D DNA nanomachine for high-efficiency amplified detection of microRNA, Talanta 240 (2022) 123219.

[34] L.N. Huang, Z.J. Zhong, Q.J. Lu, F. Chen, L.L. Xie, C.Y. Wu, Y.Y. Zhang, Simple enzyme-free biosensor for highly sensitive and selective detection of miR-21 based on multiple signal amplification strategy, J. Anal. Test. 6 (2022) 36-43.

[35] H. Kim, S. Kang, K.S. Park, H.G. Park, Enzyme-free and label-free miRNA detection based on target-triggered catalytic hairpin assembly and fluorescence enhancement of DNA-silver nanoclusters, Sens. Actuator B-Chem. 260 (2018) 140-145.
